# Exposure and risk assessment for agricultural workers during chlorothalonil and flubendiamide treatments in pepper fields

**DOI:** 10.1038/s41598-024-55172-9

**Published:** 2024-03-04

**Authors:** Deuk-Yeong Lee, Jong-Wook Song, Ji-Young An, Yeong-Jin Kim, Jong-Su Seo, Jong-Hwan Kim

**Affiliations:** Environmental Safety-Assessment Center, Korea Institute of Toxicology (KIT), Jinju, 52834 Republic of Korea

**Keywords:** Agricultural workers, Acceptable operator exposure level, Chlorothalonil, Flubendiamide, Personal protective equipment, Risk assessment, Environmental sciences, Occupational health

## Abstract

Pesticides are indispensable tools in modern agriculture for enhancing crop productivity. However, the inherent toxicity of pesticides raises significant concerns regarding human exposure, particularly among agricultural workers. This study investigated the exposure and associated risks of two commonly used pesticides in open-field pepper cultivation, namely, chlorothalonil and flubendiamide, in the Republic of Korea. We used a comprehensive approach, encompassing dermal and inhalation exposure measurements in agricultural workers during two critical scenarios: mixing/loading and application. Results revealed that during mixing/loading, dermal exposure to chlorothalonil was 3.33 mg (0.0002% of the total active ingredient [a.i.]), while flubendiamide exposure amounted to 0.173 mg (0.0001% of the a.i.). Conversely, dermal exposure increased significantly during application to 648 mg (chlorothalonil) and 93.1 mg (flubendiamide), representing 0.037% and 0.065% of the total a.i., respectively. Inhalation exposure was also evident, with chlorothalonil and flubendiamide exposure levels varying across scenarios. Notably, the risk assessment using the Risk Index (RI) indicated acceptable risk of exposure during mixing/loading but raised concerns during application, where all RIs exceeded 1, signifying potential risk. We suggest implementing additional personal protective equipment (PPE) during pesticide application, such as gowns and lower-body PPE, to mitigate these risks.

## Introduction

Pesticides are vital in reducing crop losses and improving yields and food quality by controlling diseases, pests, and weeds. However, due to the inherent toxicity of pesticides, human exposure remains a major concern^1–6^. Agricultural workers may be at risk of dermal and inhalation exposure from the use of pesticide products, which can lead to potential health complications^7–9^. The workers are exposed to pesticides across various scenarios, including mixing/loading, applying pesticides during agricultural activities, re-entering fields after spraying, and harvesting crops^2,10–12^. The predominant routes of exposure are dermal and inhalation^7,11,13,14^. Pesticide exposure is determined by several factors, including the type of agricultural work, working time, duration of contact with pesticides, field conditions, formulation, and spraying equipment^11,12,15,16^. Hence, conducting pesticide exposure assessments for agricultural workers under real field conditions is crucial.

The assessment of dermal exposure in agricultural workers can be conducted through various methods, including patch application and whole-body dosimetry (WBD)^10,16–18^. These approaches entail the collection of samples using different types of clothing or materials to extract pesticides before they contact the skin and are absorbed. Alternatively, the pesticides can be removed from the skin surface by washing the skin with a suitable solvent^19–26^. The face is a crucial area of exposure to pesticides via the eyes, mouth, and nose^11,16^. This can be achieved through various methods, including wiping, rinsing, and patch application^9,27,28^. Pesticide exposure to hands can be assessed through the use of gloves or the rinse/wash and wipe methods^20,23,28^. Traditionally, the measurement of respiratory exposure in agricultural workers involved connecting solid adsorbents like XAD-2 to personal air pumps or attaching glass fiber filters in front of solid adsorbents. However, due to the complex steps associated with these methods (e.g., cutting and connecting glass tubes for adsorbents), their application has remained a challenge^16^. To the enhance safety and efficiency of respiratory exposure, the Institute of Occupational Medicine (IOM) recently developed samplers that internally incorporate glass fiber filters. These samplers have been widely adopted for respiratory exposure measurements due to their user-friendly design and high trapping efficiency^10,11,16^. Previous studies have examined pesticide exposure levels among agricultural workers, focusing on dermal and inhalation routes and employing sampling by specific body parts, such as gloves, hands, face, and coveralls^8,10,16,18^. Additionally, the risk assessment of pesticide exposure in agricultural workers has been reported using the risk index (RI)^9,10,16,29^. RI is a widely accepted indicator for evaluating environmental risk due to its clarity and ease of interpretation^10,29^. To calculate the RI for agricultural workers, their dermal and inhalation exposures are taken into account, along with the Acceptable Operator Exposure Level (AOEL). An RI less than one indicates an acceptable risk of exposure, whereas an RI greater than one is indicative of an unacceptable risk.

Pepper (*Capsicum annuum* L.) is a globally consumed vegetable. It is cherished for its distinctive flavor and vibrant color, which has culminated in pepper products being an important part of many cuisines^30–32^. In the Republic of Korea, due to its substantial consumption, pepper cultivation is a highly profitable venture^32,33^. Specifically, pepper cultivation is largely conducted in open fields; pepper fields covering 80% of the total cultivation area in the Republic of Korea (specifically 33,373 out of 37,761 hectares)^32^. Due to the widespread nature of pepper cultivation in Korea, pests, including insects, fungi, and weeds, continue to be a serious threat to high-quality agricultural yields^33–36^. In the Republic of Korea, anthracnose and oriental tobacco budworm are recurring problems in pepper cultivation, and fungicides and insecticides are used annually to control them^37^. Chlorothalonil is a registered fungicide for controlling anthracnose, and flubendiamide is a registered insecticide for controlling oriental tobacco budworm in peppers^10,34,38^. Flubendiamide exhibits minimal toxicity through the oral, dermal, and inhalation routes. In contrast, chlorothalonil is mostly non-toxic in the context of oral or dermal exposure, but it exhibits moderate toxicity when inhaled^39,40^. Chlorothalonil and flubendiamide are widely used in pepper field in Republic of Korea^34^. However, there are few studies on the exposure of agricultural workers to these pesticides in the pepper field. Thus, it is crucial to evaluate the exposure of agricultural workers to chlorthalonil and flubendiamide to assess their potential risk.

The primary objective of this study was to evaluate the exposure and associated risks faced by agricultural workers (mixer/loader and applicator) engaged in open-field pepper cultivation, specifically concerning chlorothalonil and flubendiamide using the WBD method (for dermal exposure) and IOM sampler (for inhalation exposure). The exposure experiments were conducted through the lens of two distinct scenarios: mixing/loading and application.

## Results and discussion

### Method validation

The method limit of quantitation (MLOQ) for chlorothalonil and flubendiamide was determined to be 0.005 mg kg^−1^ in all exposure matrices. The instrumental analysis demonstrated good repeatability, with coefficients of variation (CVs) for peak area falling within the range of 7.6–8.4% for chlorothalonil and 1.3–1.8% for flubendiamide. Similarly, the CVs for retention time were notably low, ranging from 0.92 to 1.38% for chlorothalonil and 0.45–1.33% for flubendiamide. The linear relationships for chlorothalonil and flubendiamide spanned from 0.0025 mg kg^−1^ to 0.25 mg kg^−1^ and 0.005 mg kg^−1^ to 0.5 mg kg^−1^, respectively, with correlation coefficients (R^2^) exceeding 0.995. These linearities were deemed acceptable for the purpose of quantification. The recovery rates for chlorothalonil from various exposure matrices ranged from 73.6 to 113%, with corresponding CVs spanning from 0.5 to 17.1% (refer to Table S1). Similarly, the recoveries of flubendiamide from various exposure matrices fell within the range of 81.3–112%, accompanied by CVs ranging from 0.5 to 17% (refer to Table S2). Field recoveries of chlorothalonil and flubendiamide ranged from 85.6 to 110% and 93.7–105%, respectively, with CVs in the range of 3.4–7.0% for chlorothalonil and 2.9–9.6% for flubendiamide (refer to Table S1 and Table S2).

### Exposure characteristics of chlorothalonil during mixing/loading and application

**Figure 1:**
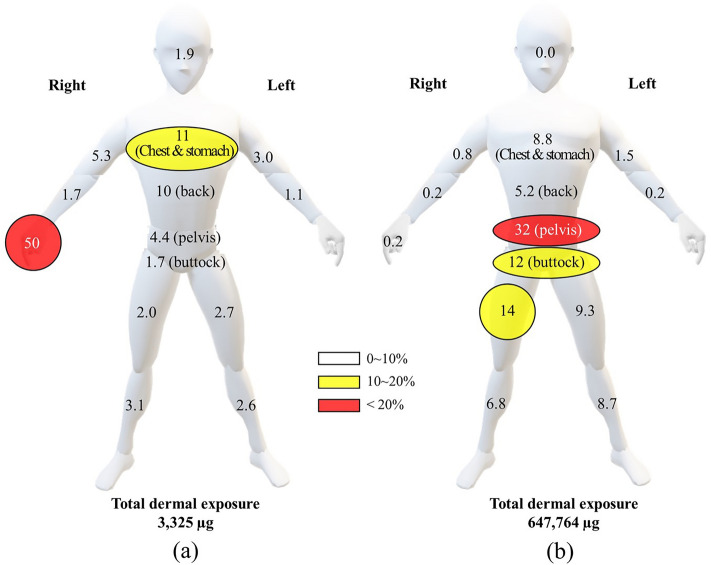
Exposure distribution pattern (%) of mixing/loading (**a**) and application (**b**) chlorothalonil by body part in agricultural workers.

In this study, we employed the WBD method to assess dermal exposure and the distribution patterns throughout the entire body of agricultural workers during the mixing/loading and application phases^8^. Table 1 and Table S3 presents data on dermal and inhalation exposure and distribution specifically for chlorothalonil. During the mixing/loading process, the dermal exposure on the entire clothing surface amounted to 3.33 mg, which equates to a mere 0.0002% of the total active ingredient (a.i.) present during mixing/loading. These findings are consistent with reported ratios of dermal exposure to total a.i. during mixing/loading, ranging from 0.0003 to 0.59% in previous studies^14,16,17,27,41–45^. Distribution analysis of worker exposure by body part during mixing and loading revealed that the highest exposure occurred on the hands, accounting for 50% of the total exposure. The chest and stomach followed at 11%, with the back at 10% (see Fig. 1). This pattern of exposure can be attributed to the direct contact of workers with the pesticide while tearing the pouch and pouring the wettable powder (WP) formulation into the mixing reservoir to create a suspension^10,27,46^. Similar distribution patterns have been reported in previous studies for various pesticides, ranging from 19.0 to 90.4% exposure on the hands^10,17,27,41,42^. Additionally, exposure distribution to the upper body (excluding the hands) accounted for 33% of the total exposure (refer to Table 1). This can be attributed to wind dispersal of the WP powder during mixing/loading, as previously documented^27,44^. Given that inner clothing is in direct contact with the skin, measuring the quantities of pesticide on innerwear is crucial as it represents a potential major dermal exposure route^9,16^. In this study, dermal exposure on inner clothing during mixing/loading was low, ranging from 0.003 to 0.02 mg. Determining the penetration rates for clothing and gloves aimed to identify body parts most susceptible to contamination due to frequent contact between outer and inner clothing^41^. Clothing penetration rates varied by body part, ranging from 0.6 to 8.9%, while the penetration rate to the hand was determined to be 3.4%. With regard to WBD, it was established that the dermal absorption of pesticides (both solid and liquid) during mixing and application is 10%^47,48^. During the mixing and loading process, inhalation exposure was measured at 0.001 mg, constituting a mere 0.0002% of the total exposure and an exceptionally low 6.9 × 10^−8^% of the total a.i. present during mixing/loading.

**Table 1:** Dermal and inhalation exposure of chlorothalonil during agricultural work in pepper field.

Body part	Exposure amount (µg)^a^							
	Mixing/loading				Application			
	Inner	Outer	Total	Distribution (%)	Inner	Outer	Total	Distribution (%)
Head	62.0	–	62	1.9	21.7	–	21.7	0.0
Chest & stomach	20.2	336	356	11	750	56,403	57,153	8.8
Back	10.8	318	329	10	481	33,416	33,897	5.2
Left upper arm	7.00	92.9	101	3.0	1,304	8,293	9,597	1.5
Right upper arm	7.16	170	177	5.3	251	5,255	5,506	0.8
Left forearm	2.82	34.8	37.6	1.1	174	1,377	1,551	0.2
Right forearm	4.21	51.3	55.5	1.7	142	1,001	1,142	0.2
Hands	9.95	1,645	1,655	50	25.4	1,105	1,130	0.2
Upper body	124	2,648	2,773	83	3,149	107,152	109,999	17
Pelvis	2.95	145	148	4.4	1,928	202,608	204,536	32
Buttocks	2.86	54.0	56.9	1.7	2,496	77,245	79,741	12
Left thigh	4.26	85.3	89.6	2.7	4,689	55,404	60,093	9.3
Right thigh	3.47	64.2	67.6	2.0	4,763	88,341	93,104	14
Left shin	7.71	79.1	86.8	2.6	1,338	54,843	56,180	8.7
Right shin	6.26	96.9	103	3.1	523	43,589	44,137	6.8
Lower body	27.5	524	552	17	15,736	522,029	537,790	83
Dermal exposure. total	152	3,172	3,325	100	18,886	629,181	647,764	100
Inhalation exposure	1.1		1.1	0.0	1.2		1.2	0.0
Total			3,325				647,765	

^a^The 75th percentile of the amount of exposure to agricultural workers.

During the application phase, the total dermal exposure on the entire clothing surface was quantified at 648 mg, accounting for 0.037% of the total a.i. present during application. This percentage aligns with the range of 0.003% to 0.066% reported in previous studies^17,27,41,44,45^. The distribution of worker exposure by body part during application indicated that the highest exposure occurred on the pelvis, constituting 32% of the total exposure, followed by the thigh at 24%, shin at 15%, and buttocks at 12% (see Fig. 1). Exposure to the hands was relatively lower at 0.2% compared to that at other body parts. This distribution pattern for chlorothalonil during application closely resembled findings reported by Choi and Kim in their research on thiophanate-methyl in pepper fields^44^. In pepper fields, characterized by high plant density and closely spaced rows, workers frequently interact with crops. This pattern is consistent with previous research on Korean cabbage, rice, and pepper, where body parts at the same height as the crops showed higher exposure distribution^8,25,41,44,49^. Dermal exposure on inner clothing during application ranged from 0.025 to 4.763 mg. The penetration rate to the hand was found to be 1.4%, whereas clothing penetration rates varied across body parts, ranging from 0.9 to 14%. Notably, the left upper arm (14%) and left forearm (12%) exhibited higher clothing penetration rates, in line with the commonly used default value of 10%^17,47,48^. In terms of inhalation exposure during application, the recorded amount was 0.001 mg (refer to Table 1). This inhalation exposure represented a mere 0.002% of the total exposure and an exceedingly low 6.9 × 10^−8^% of the total a.i. during application. These figures are consistent with findings from previous studies, where inhalation exposure ranged from 0.001 to 0.115% of the total exposure and from 7.6 × 10^−7^ to 4.9 × 10^−5^% of the total a.i. during application^10,27,41,42^.

### Exposure characteristics of flubendiamide during mixing/loading and application

**Figure 2:**
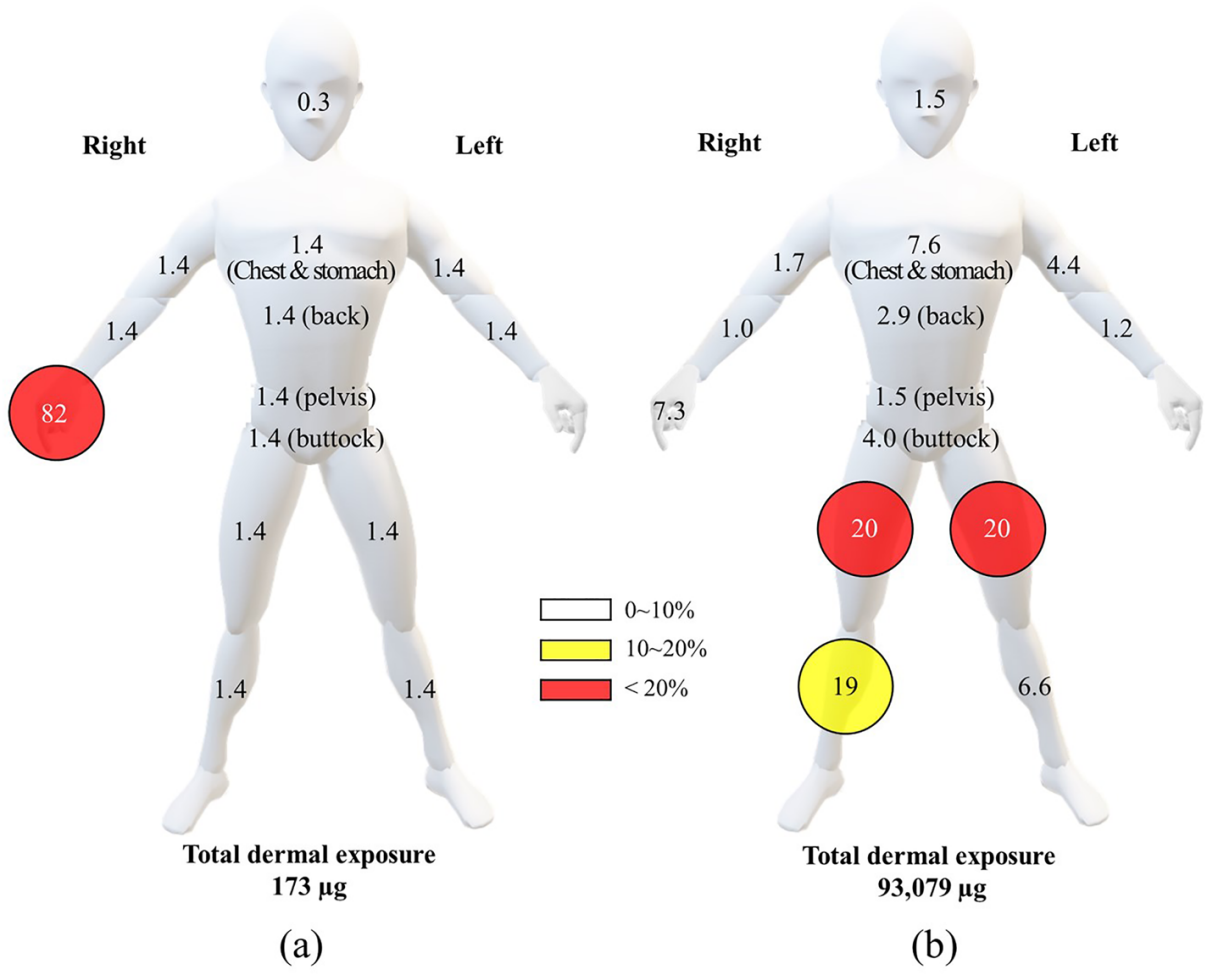
Exposure distribution pattern (%) of mixing/loading (**a**) and application (**b**) flubendiamide by body part in agricultural workers.

The dermal exposure amount of flubendiamide on whole clothing during mixing/loading was 0.173 mg (refer to Table 2 and Table S4), which was 0.0001% of the total a.i. in mixing/loading. The ratios of dermal exposure to the total a.i. in mixing/loading were lower than those reported in previous studies (0.0003–0.59%)^14,16,17,28,41–45^. Previous studies using liquid formulations (emulsifiable and soluble concentrates) showed large variations in dermal exposure rates relative to the total a.i. of mixing and loading, ranging from 0.0007 to 0.59%. In the current study, it was 0.0001% (suspension concentrate, SC)^44^. Dermal exposure during mixing/loading before spraying is influenced by field conditions rather than crop differences and mainly affects the hands. Therefore, wearing protective gloves during mixing and loading is recommended. The distribution of worker exposure by body part during mixing/loading was the highest for hands (82%) (see Fig. 2). The distribution pattern on the hands for chlorothalonil (see Fig. 1) was similar to that of previous studies^9,10,28,41,43^. This trend can be attributed to direct contamination during mixing and loading. Therefore, we recommended that agricultural work should wear protective gloves while mixing and loading to minimize dermal exposure, particularly on the hands^28,43,44^. Dermal exposure to the inner clothing during mixing/loading was below LOQ (< 0.005 mg kg^−1^); the penetration rates to various body parts and hands were below 2.5%. The amount of inhalation exposure during mixing and loading was 0.00027 mg, representing 0.2% of the total exposure and 1.9 × 10^−7^% of the total a.i. during mixing/loading. The inhalation exposure amount was approximately 100-fold lower than that of chlorothalonil (refer to Tables 1 and 2). This could be related to the dispersion caused by the formulation during mixing/loading.

**Table 2:** Dermal and inhalation exposure of flubendiamide during agricultural work in pepper field.

Body part	Exposure amount (µg)^a^							
	Mixing/loading				Application			
	Inner	Outer	Total	Distribution (%)	Inner	Outer	Total	Distribution (%)
Head	0.50	–	0.50	0.3	1,369	–	1,369	1.5
Chest & stomach	1.25	1.25	2.50	1.4	578	6,509	2,704	7.6
Back	1.25	1.25	2.50	1.4	602	2,126	7,110	2.9
Left upper arm	1.25	1.25	2.50	1.4	460	3,611	4,071	4.4
Right upper arm	1.25	1.25	2.50	1.4	386	1,218	1,604	1.7
Left forearm	1.25	1.25	2.50	1.4	367	773	1,140	1.2
Right forearm	1.25	1.25	2.50	1.4	364	529	893	1.0
Hands	2.50	140	143	82	2.50	6,772	6,774	7.3
Upper body	10.5	148	158	91	4,128	21,537	26,226	28
Pelvis	1.25	1.25	2.50	1.4	174	1,253	1,426	1.5
Buttocks	1.25	1.25	2.50	1.4	893	2,797	3,690	4.0
Left thigh	1.25	1.25	2.50	1.4	3,583	15,038	18,620	20
Right thigh	1.25	1.25	2.50	1.4	3,121	15,803	18,924	20
Left shin	1.25	1.25	2.50	1.4	238	5,859	6,097	6.6
Right shin	1.25	1.25	2.50	1.4	4,205	13,667	18,096	19
Lower body	7.50	7.5	15.0	8.6	12,213	54,415	66,853	72
Dermal exposure. total	18.0	155	173	100	16,341	75,953	93,079	99.1
Inhalation exposure	0.025		0.025	0.0	66.2		66.2	0.1
Total			173				93,360	

^a^The 75th percentile of the amount of exposure to agricultural workers.

The dermal exposure on whole clothing during application was 93.1 mg (refer to Table 2), representing 0.065% of the total a.i. during application. This percentage is similar to the previously reported range of 0.003–0.066%^17,27,41,44,45^. The distribution of worker exposure by body part during application showed the highest exposure on the thigh (40%), followed by the shin (26%), chest and stomach (7.6%), and hand (7.3%) (see Fig. 2). The distribution on lower body parts (thigh, shin, pelvis, and buttocks) for flubendiamide during application was 72% (refer to Table 2), which was similar to the distribution on lower body parts for chlorothalonil (83%, Table 1). This similarity may be linked to field conditions, such as crop height and the degree of contact between workers and crops. Crop height in the flubendiamide field was lower than that in the chlorothalonil field (refer to Table S5), which may explain the high exposure of the thigh to flubendiamide^25,44^. During application, dermal exposure on inner clothing ranged from 0.003 to 4.205 mg. Clothing penetration rates of the upper body ranged from 9.2 to 69%, whereas the penetration rate to the hand was 0.04%, and lower body penetration ranged from 4 to 32%. The field conditions for flubendiamide, including field area and spray volume, were more extensive than those for chlorothalonil (refer to Table S5). Therefore, the increased frequency of contact between workers and crops may result in a higher rate of flubendiamide penetration. The right arm demonstrated the highest penetration (upper arm 32%, forearm 69%) since the applicator handles the nozzle with the right arm, leading to more contact with the crop during application^20,43^. Applicators may use additional PPE such as arm sleeves and gowns to decrease arm exposure. The inhalation exposure amount during application was 0.066 mg (refer to Table 2), which was 0.1% of the total exposure and 4.7 × 10^−5^% of the total a.i. of during application. These inhalation exposure rates were higher than those reported in previous studies (0.001–0.115% of total exposure and 7.6 × 10^−^^7^ to 4.9 × 10^−5^% of the total a.i. during application^10,27,41,42^.

### Risk assessment of chlorothalonil and flubendiamide

In this study, we calculated RIs as part of the risk assessment for chlorothalonil and flubendiamide among agricultural workers^29,41^. RIs greater than 1 indicate the presence of a potential risk. We considered exposure to inner clothing as actual dermal exposure (ADE), with the default dermal adsorption rate of 10%^48^. Similarly, inhalation exposure was also considered ADE. The AOELs for chlorothalonil and flubendiamide were established at 0.009 mg kg_bw_^−1^day^−1^ and 0.006 mg kg_bw_^−1^day^−1^, respectively^47^. Based on these values, the RIs for chlorothalonil and flubendiamide were computed as follows: 4.3 × 10^−2^ and 0.7 × 10^−3^ for mixing/loading, and 4.6 and 5.8 for application, respectively (refer to Table 3). Agricultural workers involved in mixing/loading can generally be considered acceptable risk, as the RIs for both pesticides were well below 1. However, it is important to emphasize the significance of wearing protective gloves to protect the hands, given the high exposure observed (see Figs. 1 and 2). Chlorothalonil and flubendiamide may pose a risk to applicators, as their respective RIs exceed 1. If a single agricultural worker is responsible for both mixing/loading and application, the RIs for chlorothalonil and flubendiamide are 4.7 and 6.0, respectively. This approach to pesticide application greatly increases a worker’s risk of exposure. In a previous study, the combination of polyester/cotton coveralls and body gowns demonstrated a high level of protection (98.7%), indicating its effectiveness in safeguarding the body^28^. Therefore, to mitigate the risk associated with chlorothalonil and flubendiamide for applicators, additional PPE such as gowns or lower body protection could be employed during application to shield workers from sprayed pesticides and potential contact with crops^28,29^. In present study, we assessed agricultural workers’ dermal and inhalation exposure. However, research on the internal pesticide exposure of agricultural workers during various farming activities remains limited, which warrants further investigation.

**Table 3:** Risk assessment of agricultural workers of chlorothalonil and flubendiamide on pepper field.

Pesticide	Chlorothalonil		Flubendiamide	
Scenarios	Mixing/loading	Application	Mixing/loading	Application
Body weight (kg)	70	70	70	70
AOEL (mg kg_bw_^−1^ day^−1^)^a^	0.009	0.009	0.006	0.006
AF (%)^b^	10	10	10	10
ADE (μg day^−1^)^c^	152	18,886	18	16,341
AIE (μg day^−1^)^d^	12.1	12.7	0.27	701
RI	4.3 $\times$ 10^−2^	4.6	0.7 $\times$ 10^−3^	5.8

^a^AOEL (acceptable operator exposure level); ^b^AF (skin absorption rate); ^c^ADE (actual dermal exposure); ^d^AIE (actual inhalation exposure).

## Conclusions

This study employed the WBD method to evaluate the exposure and risk of chlorothalonil and flubendiamide among agricultural workers in pepper fields in the Republic of Korea. The assessment considered two distinct scenarios: mixing/loading and application. In the mixing/loading scenario, the hands had the highest exposure to chlorothalonil and flubendiamide. Conversely, in the application scenario, agricultural workers experienced higher exposure to these pesticides in the lower part of their bodies, including the pelvis and thighs. Importantly, inhalation exposure in both scenarios remained below 1% of the total exposure for agricultural workers. RIs were calculated for dermal and inhalation exposure, and the results indicated that for both chlorothalonil and flubendiamide, the RIs during mixing/loading were below 1, signifying the acceptable risk of this phase for workers. However, the RIs for applying chlorothalonil and flubendiamide to pepper crops exceeded 1, suggesting a potential risk to workers. To mitigate this risk, we recommend protective measures. Agricultural workers should safeguard their hands by wearing gloves during mixing/loading, where direct hand exposure is prominent. Additionally, to protect workers from potential pesticide exposure and prevent skin contact with the crops, using additional lower-body PPE during application in pepper fields is advisable. This study primarily evaluated inhalation and dermal pesticide exposures in agricultural workers. To comprehensively assess pesticide exposures in this population, further studies involving biomonitoring with urine and blood samples taken before and after work are needed, which will provide a more comprehensive understanding of the health risks associated with pesticide exposure among agricultural workers.

## Methods

### Chemicals and reagents

Analytical standards of chlorothalonil and flubendiamide were purchased from HPC Standard GmbH (Borsdorf, Germany). HPLC-grade acetonitrile, methanol, and water were purchased from Honeywell Burdick & Jackson (Morristown, NJ, USA). Reagent-grade formic acid (purity > 98%) was purchased from Sigma-Aldrich® (Merck KgaA, Darmstadt, Germany). The following pesticides were tested: 75% chlorothalonil (WP) obtained from Kyungnong (Seoul, Korea) and 20% flubendiamide (SC) purchased from HanKookSamGong (Seoul, Korea). The detergent of Aerosol® OT-75 was purchased from Jaekyu Chemical (Seoul, Korea).

### Exposure matrices of agricultural workers

The study was approved by the Institutional Review Board (IRB) in the Republic of Korea and informed consent was obtained from all participants (i.e., agricultural workers in pepper cultivation). The field trials in North Gyeongsang Province, Republic of Korea, were permitted by an organization, the National Institute of Agricultural Sciences, certified by the Rural Development Administration of the Republic of Korea to carry out trials and collect samples. These trials were conducted in accordance with the relevant institutional, national, and international Good Laboratory Practice guidelines and legislation^48–50^.

The dermal exposure of agricultural workers was measured using whole-body dosimetry (WBD). Dermal exposure of each body part was measured while wearing outer clothing (65/35, polyester/cotton, Uniseven, Seoul, Korea) and inner clothing (100% cotton, TRY®, Ssangbangwool, Seoul, Korea). Head and hand exposure were measured using gauze (10 × 10 cm) and nitrile gloves. To estimate the amount of pesticide, the head (face and neck) was wiped with 0.01% Aerosol® OT-75 detergent-soaked gauze and then analyzed. The hands were thoroughly washed with a 0.01% aqueous detergent solution while wearing nitrile gloves. Subsequently, the gloves were removed, and the washing procedure was repeated. The washing solutions of gloves and hands were analyzed to estimate the amount of pesticide on the hands. Inhalation exposure was measured using a personal air pump (GilAir-3, Sensidyne, Clearwater, FL, USA) and an IOM sampler (SKC, Eighty Four, PA, USA) with a glass fiber filter (25 mm, SKC, Eighty Four, PA, USA).

### Field trials and sampling

All field trials were conducted in the pepper-growing area located in North Gyeongsang Province in the Republic of Korea. Chlorothalonil 75% WP and flubendiamide 20% SC commercial products were diluted and mixed 600 and 2000 times, respectively^38^. A power sprayer was used to apply the pesticide solutions to pepper fields. The pesticides are sprayed using conventional pesticide application methods on pepper fields. The amount of active ingredient used per 1000 m^2^ for chlorothalonil was 0.162–0.378 kg a.i 1000 m^−2^, while for flubendamide it was 0.013–0.033 kg a.i 1000 m^−2^ (refer to Table S5). The pesticides were applied in July and September—the months when they are typically sprayed on pepper fields. Field trials were conducted for chlorothalonil and flubendiamide using two mixing/loading and application scenarios, each replicated ten times (see Fig. S1). The mixing/loading scenario involved one mixer/loader tested ten times, while the application scenario entailed ten applicators tested once. All agricultural workers wore personal protective equipment (PPE) such as outer and inner clothing, nitrile gloves, and inhalation exposure meters while mixing/loading and applying the pesticide. The air pump flow was calibrated and set to 2 L min^−1^ prior to testing. A mixing/loading worker mixed the pesticide suspension (1000 L) for 30 min to prepare the pesticide application. Applicators then applied the solutions to the pepper field using a hand-held sprayer with a hose and nozzle. Detailed information on the field, application, and climatic conditions is provided in the Supporting Information Table S5. After the mixing/loading and applying the pesticide, the gloves and hands were cleaned with 0.5 L of 0.01% detergent solution. The head (including face and neck) was wiped twice with gauze soaked in 4 mL of 0.01% detergent solution. All of the workers clothing (outer and inner) was taken off to avoid cross-contamination and divided into 11 pieces (see Fig. S1). After switching off the personal air pump, the fiberglass filter was removed from the IOM cassette. The sample was then stored at − 20 °C prior to sample extraction and instrumental analysis.

### Analytical sample preparation and instrumental analysis

To analyze chlorothalonil residues on operator exposure matrices, inner and outer clothing were extracted with a 500 mL solution of 0.1% formic acid in acetonitrile. Gauze matrices were extracted with 50 mL, whereas glass fiber filters were treated with 10 mL of the same solution. The extract was shaken in a vertical shaker (SR-2DW, TAITEC, Koshigaya, Japan) at 300 rpm for 1 h and then filtered through a syringe filter (0.22 μm, PTFE, Whatman, Maidstone, UK). Hand wash solutions were checked for residues of chlorothalonil on gloves and hands. The hand and glove wash solutions were extracted using liquid–liquid extraction with dichloromethane, concentrated, and redissolved in acetonitrile for analysis. For the quantitative analysis of chlorothalonil, the separation was conducted using a DB-5MS UI column (30 m × 0.25 mm, 0.25 µm, Agilent Technologies, Santa Clara, CA, USA), and the analysis was performed by GC–MS/MS (SCION TQ, Bruker, Billerica, MA, USA). The detailed instrument conditions are provided in Table S6.

Extraction was performed using methanol to analyze flubendiamide residues on operator exposure matrices (inner and outer clothing, gauze, and glass fiber filter). For the analysis of flubendiamide on the hand and glove, each wash solution was analyzed after it was centrifuged 300 rpm for 1 h. The extraction procedure was carried out as described above. For the quantitative analysis of flubendiamide, the separation was carried out using a Poroshell 120 EC-C18 column (2.1 × 100 mm, 2.7 µm, Agilent Technologies, Santa Clara, CA, USA), and the analysis was performed by LC–MS/MS (Agilent 6420 series, Agilent Technologies, Santa Clara, CA, USA). The detailed instrument conditions are provided in Table S7.

### Method validation for quantitative analysis of the pesticide

For the preparation of a 1000 mg kg^−1^ stock solution, the standard solution of chlorothalonil was diluted with 0.1% formic acid in acetonitrile, and the standard solution of flubendiamide was diluted with methanol. For each body part, a piece of inner and outer clothing (30 × 30 cm), hand and glove washing solution, glass fiber filter, and gauze were extracted as explained in Section “Risk assessment of chlorothalonil and flubendiamide”. The extracts were then processed with the solvent standard to produce a matrix-matched standard solution in the range of 0.005–0.5 mg kg^−1^ for flubendiamide and 0.0025–0.25 mg kg^−1^ for chlorothalonil, respectively. The method validation is conducted according to previous research^10,17^. The MLOQ was calculated using the instrument limit of quantitation and injection volume, solvent volume of extract, and the standard solution^10^. To verify the instrument’s repeatability, various concentrations of standards (MLOQ and 10 MLOQ) were analyzed seven times by LC–MS/MS for flubendiamide and GC–MS/MS for chlorothalonil. The linearity of the calibration curve was confirmed using the various matrix-matched standards. The recovery test was carried out by spiking three levels (MLOQ, 10MLOQ, 100MLOQ) of standard solutions to the control matrices. In-field recovery testing was conducted, with each sample treated to a 100 MLOQ standard under field conditions, exposed to the environment, and then analyzed. All analyses were carried out in triplicate.

### Exposure and risk assessment of agricultural workers

The dermal exposure amount (μg) to chlorothalonil and flubendiamide was calculated by considering the residues on clothing based by body part and the amount of solvent used for their extraction. Exposure amounts for gloves and hands were calculated by considering the residue on the wash solution and the amount of solvent used for extraction^21^. If the detection value is below the LOQ, half of the LOQ value was used^16,41^. Head exposure was assumed to be twice the amount of gauze residue that was used to wipe the face and neck^21^. Penetration rates were estimated by considering combined exposure to hands and gloves, while clothing penetration rates were estimated by considering combined exposure to inner and outer clothing^9,17^. To determine the inhalation exposure (μg), a respiratory rate of 1270 L h^−1^ was assumed for an adult male in Korea^17,41,50^. The inhalation exposure to chlorothalonil and flubendiamide was extrapolated from the flow rate of the air pump (2 mL min^−1^) and the residue of the glass fiber filter.

The exposure assessment was based on the 75th percentile of 10 replicates^51,52^. In the Republic of Korea, the power sprayer is set to spray one hectare per day with a volume of 1500 L per hectare for pepper^16,20,47^. Thus, extrapolated to 1500 L of spray volume, we assessed the risk to agricultural workers. The calculation of the Retention Index (RI) was performed using Eq. (1) ^29,48^.

1 $$RI=\frac{\left(ADE\times AF\right)+AIE}{AOEL\times body\, weight\, (kg)}$$

ADE (μg) was inner clothing exposure amount, AIE (μg) was the actual inhalation exposure, and the body weight was assumed to be 70 kg. In Republic of Korea, the dermal absorption factor (AF) for liquid and solid formulations among agricultural workers is established at 10%^48^. Additionally, the AOEL for chlorothalonil and flubendiamide is set at 0.009 mg kg_bw_^−1^ day^−1^ and 0.006 mg kg_bw_^−1^ day^−1^, respectively^49^.

### Ethics approval and Consent to participate

Informed consent was obtained from all individual participants included in the study. The authors affirm that human research participants provided informed consent for publication of the images in Figure(s) S2.

### Supplementary Information

Supplementary Information.

## Data Availability

All data generated or analyzed during this study are included in this published article.
