# Author Correction: Exposure and risk assessment for agricultural workers during chlorothalonil and flubendiamide treatments in pepper fields

**DOI:** 10.1038/s41598-024-59601-7

**Published:** 2024-04-18

**Authors:** Deuk-Yeong Lee, Jong-Wook Song, Ji-Young An, Yeong-Jin Kim, Jong-Su Seo, Jong-Hwan Kim

**Affiliations:** Environmental Safety-Assessment Center, Korea Institute of Toxicology (KIT), Jinju, 52834 Republic of Korea

Correction to: *Scientific Reports* 10.1038/s41598-024-55172-9, published online 04 March 2024

The original version of this Article contained errors in Table 3, were the values in the “ADE (μg day^−1^)^c^” row were incorrect for chlorothalonil and flubendiamide.

The correct and incorrect values appear below.

Incorrect:
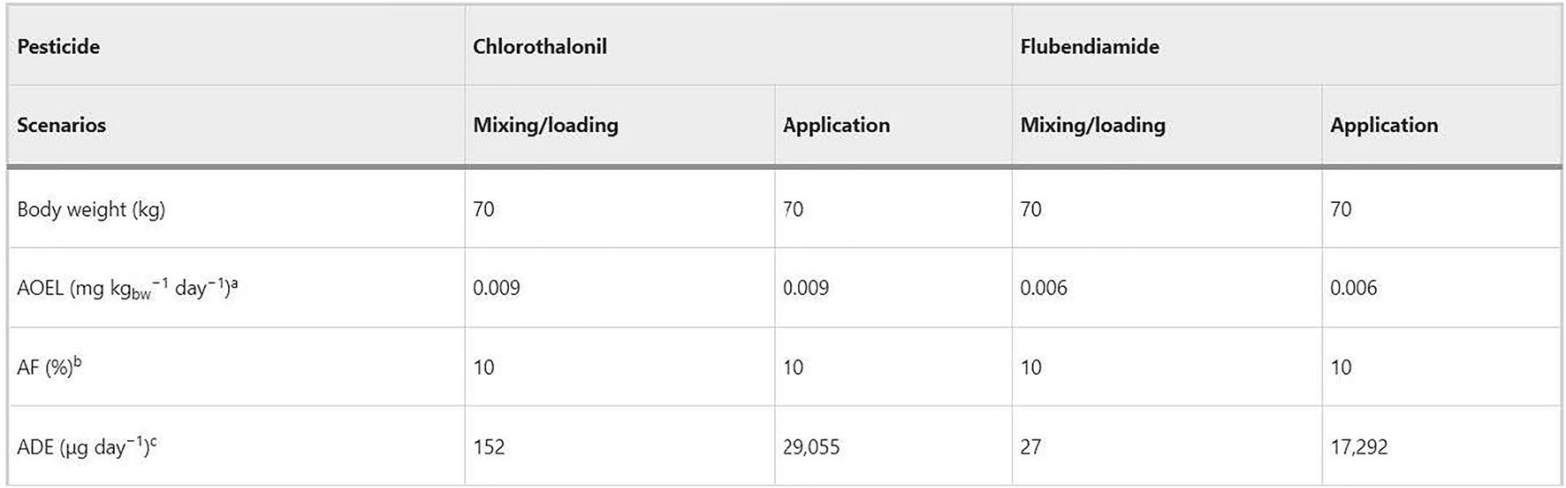


Correct:

**Table:** 

Pesticide	Chlorothalonil		Flubendiamide	
Scenarios	Mixing/loading	Application	Mixing/loading	Application
Body weight (kg)	70	70	70	70
AOEL (mg kg_bw_^−1^ day^−1^)^a^	0.009	0.009	0.006	0.006
AF (%)^b^	10	10	10	10
ADE (μg day^−1^)^c^	152	18,886	18	16,341

The original Article has been corrected.

