# Supplementary material for: Exposure and risk assessment for agricultural workers during chlorothalonil and flubendiamide treatments in pepper fields
Source: Sci Rep. 2024 Mar 4;14:5338. doi: 10.1038/s41598-024-55172-9 (PMC10912086; doi:10.1038/s41598-024-55172-9)
Supplement: Supplementary file 1 — Supplementary Information. [file 41598_2024_55172_MOESM1_ESM.docx]

**Supporting information**

**Exposure and risk assessment for agricultural workers during chlorothalonil and flubendiamide treatments in pepper fields**

Deuk-Yeong Lee^1^, Jong-Wook Song^1^, Ji-Young An^1^, Yeong-Jin Kim^1^, Jong-Su Seo^1^, and Jong-Hwan Kim^1,*^

*^1^Environmental Chemistry Research Group, Korea Institute of Toxicology, Jinju, 52834, Republic of Korea*

**Table S1.** Recoveries, CVs and MLOQ of the chlorothalonil in exposure matrices

| Matrix | Fortification level | Recovery (%) | CV (%) | MLOQ (mg kg^‑1^) |
| --- | --- | --- | --- | --- |
| Outer clothing | MLOQ | 104 | 2.6 | 0.005 |
|  | 10MLOQ | 94.5 | 1.3 |  |
|  | 100MLOQ | 96.2 | 0.5 |  |
|  | 100MLOQ^a^ | 103 | 5.1 |  |
| Inner clothing | MLOQ | 101 | 1.2 | 0.005 |
|  | 10MLOQ | 107 | 1.3 |  |
|  | 100MLOQ | 103 | 0.6 |  |
|  | 100MLOQ^a^ | 101 | 6.2 |  |
| Gauze | MLOQ | 102 | 1.7 | 0.005 |
|  | 10MLOQ | 93.7 | 2.5 |  |
|  | 100MLOQ | 87.6 | 1.5 |  |
|  | 100MLOQ^a^ | 85.6 | 3.4 |  |
| Glove  (washing solution) | MLOQ | 73.6 | 12 | 0.005 |
|  | 10MLOQ | 92.3 | 17 |  |
|  | 100MLOQ | 113 | 10 |  |
|  | 100MLOQ^a^ | 110 | 4.0 |  |
| Hand  (washing solution) | MLOQ | 112 | 6.1 | 0.005 |
|  | 10MLOQ | 97.0 | 13 |  |
|  | 100MLOQ | 107 | 16 |  |
|  | 100MLOQ^a^ | 110 | 7.0 |  |
| Glass fiber filter | MLOQ | 110 | 2.2 | 0.005 |
|  | 10MLOQ | 97.4 | 1.5 |  |
|  | 100MLOQ | 87.6 | 1.8 |  |
|  | 100MLOQ^a^ | 86.4 | 4.4 |  |

^a^ Field recovery test

**Table S2.** Recoveries, CVs and MLOQ of the flubendiamide in exposure matrices

| Matrix | Fortification level | Recovery (%) | CV (%) | MLOQ (mg kg^‑1^) |
| --- | --- | --- | --- | --- |
| Outer clothing | MLOQ | 84.7 | 3.6 | 0.005 |
|  | 10MLOQ | 111 | 8.5 |  |
|  | 100MLOQ | 109 | 3.9 |  |
|  | 100MLOQ^a^ | 103 | 9.6 |  |
| Inner clothing | MLOQ | 86.7 | 4.8 | 0.005 |
|  | 10MLOQ | 112 | 1.8 |  |
|  | 100MLOQ | 110 | 0.8 |  |
|  | 100MLOQ^a^ | 97.1 | 4.9 |  |
| Gauze | MLOQ | 81.3 | 3.8 | 0.005 |
|  | 10MLOQ | 107 | 3.3 |  |
|  | 100MLOQ | 93.0 | 1.0 |  |
|  | 100MLOQ^a^ | 105 | 2.9 |  |
| Glove  (washing solution) | MLOQ | 86.7 | 3.5 | 0.005 |
|  | 10MLOQ | 102 | 3.9 |  |
|  | 100MLOQ | 103 | 1.1 |  |
|  | 100MLOQ^a^ | 93.7 | 6.7 |  |
| Hand  (washing solution) | MLOQ | 86.0 | 6.2 | 0.005 |
|  | 10MLOQ | 101 | 4.1 |  |
|  | 100MLOQ | 97.3 | 5.5 |  |
|  | 100MLOQ^a^ | 101 | 3.7 |  |
| Glass fiber filter | MLOQ | 90.6 | 2.1 | 0.005 |
|  | 10MLOQ | 102 | 3.3 |  |
|  | 100MLOQ | 98.6 | 1.7 |  |
|  | 100MLOQ^a^ | 97.9 | 3.9 |  |

^a^ Field recovery test

**Table S3.** The range of dermal and respiratory exposure to chlorothalonil during mixing/loading and application in a pepper field^a^

| **Body parts** | **Exposure amount (ug) [min-max (75th percentile)]** | | | |
| --- | --- | --- | --- | --- |
|  | **Mixing/Loading** | | **Application** | |
|  | **Inner** | **Outer** | **Inner** | **Outer** |
| **Head** | 30.2–84.3 (62.0) |  | 1.32–48.9 (21.7) |  |
| **Chest & stomach** | 1.25–40.7 (20.2) | 263–848 (336) | 91.1–1,098 (750) | 3,410–181,301 (56,403) |
| **Back** | 1.25–16.8 (10.8) | 191–587 (318) | 23.7–608 (481) | 2,443–56,469 (33,416) |
| **Left upper arm** | 1.25–27.7 (7.00) | 6.37–280 (92.9) | 11.1–2,089 (1,304) | 359–11,433 (8,293) |
| **Right upper arm** | 1.25–25.0 (7.16) | 170–486 (170) | 10.2–881 (251) | 547–12,441 (5,255) |
| **Left forearm** | 1.25–5.94 (2.82) | 20.3–65.4 (34.8) | 18.4–626 (174) | 388–1,680 (1,377) |
| **Right forearm** | 1.25–34.7 (4.21) | 26.9–80.5 (51.3) | 14.7–586 (142) | 466–3,273 (1,001) |
| **Hands** | 2.50–19.7 (9.95) | 455–1,699 (1,645) | 2.50–40.8 (25.4) | 53.5–1,859 (1,105) |
| **Pelvis** | 1.25–14.0 (2.95) | 257–651 (145) | 19.1–4,401 (1928) | 1,719–255,230 (202,608) |
| **Buttocks** | 1.25–27.2 (2.86) | 91.2–291 (54.0) | 44.5–3,331 (2496) | 2,230–105,478 (77,245) |
| **Left thigh** | 1.25–12.0 (4.26) | 50.8–159 (85.3) | 36.4–9,479 (4689) | 904–91,826 (55,404) |
| **Right thigh** | 1.25–14.8 (3.47) | 52.9–162 (64.2) | 22.2–7,704(4763) | 2,891–184,611 (88,341) |
| **Left shin** | 1.25–15.8 (7.71) | 68.9–240 (79.1) | 60.0–4,581 (1338) | 2,338–141,042 (54,843) |
| **Right shin** | 1.25–26.9 (6.26) | 50.1–149 (96.9) | 41.5–2,614 (523) | 6,411–66,819 (43,589) |
| **Inhalation** | 0.1–6.5 (1.1) |  | 0.18–2.3 (1.2) |  |

^a^ To estimate exposure amounts below the Limit of Quantification (LOQ), half of the LOQ value was used.

**Table S4.** The range of dermal and respiratory exposure to flubendiamide during mixing/loading and application in a pepper field^a^

| **Body parts** | **Exposure amount (ug) [min–max (75th percentile)]** | | | |
| --- | --- | --- | --- | --- |
|  | **Mixing/Loading** | | **Application** | |
|  | **Inner** | **Outer** | **Inner** | **Outer** |
| **Head** | 0.50–2.80 (0.5) |  | 430–2,667 (1,369) |  |
| **Chest & stomach** | 1.25 (1.25) | 1.25–11.5 (1.25) | 515–623 (578) | 1,609–11,998 (6,509) |
| **Back** | 1.25 (1.25) | 1.25 (1.25) | 520–638 (602) | 880–2,725 (2,126) |
| **Left upper arm** | 1.25 (1.25) | 1.25–17.0 (1.25) | 351–1,044 (460) | 489–4,160 (3,611) |
| **Right upper arm** | 1.25 (1.25) | 1.25 (1.25) | 336–402 (386) | 528–2,127 (1,218) |
| **Left forearm** | 1.25 (1.25) | 1.25 (1.25) | 344–512 (367) | 147–1,317 (773) |
| **Right forearm** | 1.25 (1.25) | 1.25 (1.25) | 336–552 (364) | 106–869 (529) |
| **Hands** | 2.50 (2.50) | 40.6–413 (140) | 2.50 (2.50) | 2,500–15,755 (6,772) |
| **Pelvis** | 1.25 (1.25) | 1.25–7.77 (1.25) | 15.5–427 (174) | 517–2,412 (1,253) |
| **Buttocks** | 1.25 (1.25) | 1.25 (1.25) | 519–1,275 (893) | 757–4,152 (2,797) |
| **Left thigh** | 1.25 (1.25) | 1.25 (1.25) | 290–8,130 (3,583) | 996–18,519 (15,038) |
| **Right thigh** | 1.25 (1.25) | 1.25 (1.25) | 285–6,536 (3,121) | 2,013–19,239 (15,803) |
| **Left shin** | 1.25 (1.25) | 1.25–5.68 (1.25) | 33.9–430 (238) | 289–8,680 (5,859) |
| **Right shin** | 1.25 (1.25) | 1.25–21.0 (1.25) | 291–7,179 (4,205) | 1,641–14,961 (13,667) |
| **Inhalation** | 0.27 (0.27) |  | 3.3–203 (66.2) |  |

^a^ To estimate exposure amounts below the Limit of Quantification (LOQ), half of the LOQ value was used.

**Table S5.** Field, application and climate conditions during the pepper field trials

| **Condition** | **Region (pesticide)** | |
| --- | --- | --- |
|  | **Uiseong–gun**  **(chlorothalonil)** | **Yeongyang–gun**  **(flubendiamide)** |
| **Field** |  |  |
| Plant height (cm) | 70–120 (95) | 60–120 (85) |
| Plantation distance (cm) | 30–40 (33.8) | 30–50 (38.4) |
| Row distance (cm) | 70–130 (99.5) | 100–140 (121) |
| Field area (m^2^) | 2,000–4,700 | 3,300–10,000 |
| **Application** |  |  |
| Applicator | Power sprayer | Power sprayer |
| Lance length (cm) | 90–112 | 80–112 |
| Nozzle type (No.) | Full cone nozzle (1) | Full cone nozzle (1) |
| Nozzle pressure (MPa) | 2.5–3.4 | 2.0–5.0 |
| Spraying volume (L/1,000 m^2^) | 130–302 | 134–334 |
| Treatment amount (kg a.i/1,000 m^2^) | 0.162–0.378 | 0.013–0.033 |
| **Climate** |  |  |
| Temperature (℃) | 25.3–33.1 (28.3) | 20.5–31.5 (25.4) |
| Relative humidity (%) | 59–91.4 (77.8) | 55.3–89.5 (74.1) |
| Wind speed (m/sec) | 0.0–1.8 (0.61) | 0.0–2.5 (1.3) |

**Table S6.** The instrumental condition of GC–MS/MS for quantitative analysis of chlorothalonil

| **Instrument** | Bruker SCION triple quadrupole (TQ) GC–MS (Bruker, Billerica, MA, USA) | | |
| --- | --- | --- | --- |
| **Injection** | 2 μL (250℃) | | |
| **Column** | DB–5MS UI (30m × 0.25 mm, 0.25 µm) | | |
| **Flow** | 1.0 mL min^–1^ (He) | | |
| **Oven condition** | Temperature (℃) | Rate (℃ min^–1^) | Hold (min) |
|  | 150 | 0 | 2 |
|  | 300 | 25 | 4 |
| **Ionization source** | Electron ionization (EI) | | |
| **Polarity** | Positive | | |
| **Source temperature** | 220℃ | | |
| **Transferline temperature** | 260℃ | | |
| **MRM** | Precursor ion (*m/z*) | Product ion  (*m/z*) | Collision energy (V) |
|  | 266 | 231 | 20 |
|  | 266 | 168 | 20 |

**Table S7.** The instrumental condition of LC–MS/MS for quantitative analysis of flubendiamide

| **Instrument** | Agilent Technologies 6420 Triple Quad LC/MS (Agilent, Santa Clara, CA, USA) | | | | | |
| --- | --- | --- | --- | --- | --- | --- |
| **Column** | Poroshell 120 EC–C18 (2.1 × 100 mm, 2.7 µm) | | | | | |
| **Flow** | 0.3 mL min­^–1^ | | | | | |
| **Mobile phase** | A : 0.1% Formic acid with 5mM ammonium formate in water  B : 0.1% Formic acid with 5mM ammonium formate in methanol | | | | | |
|  | Time (min) | | %A | | %B | |
|  | 5 | | 20 | | 80 | |
| **Injection volume** | 4 μL | | | | | |
| **Ionization source** | Electrospray ionization (ESI) | | | | | |
| **Polarity** | Negative | | | | | |
| **Source temperature** | 150℃ | | | | | |
| **Capillary voltage** | 4000 V | | | | | |
| **Gas temperature** | 325℃ (N_2_, 360 L hr^–1^) | | | | | |
| **MRM** | Precursor ion (*m/z*) | Product ion (*m/z*) | | Fragment voltage (V) | | Collision energy (V) |
|  | 681 | 273.9 | | 200 | | 8 |
|  | 681 | 254.2 | | 200 | | 18 |

**
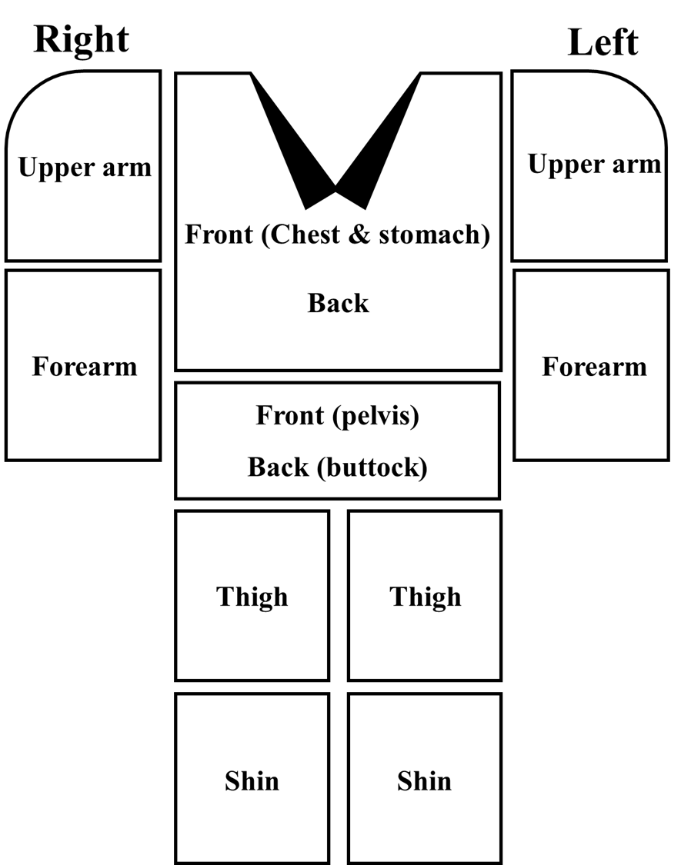
**

**Fig S1.** Sampling of agricultural workers clothing (outer and inner) for the calculated exposure amount by part of the body.

**
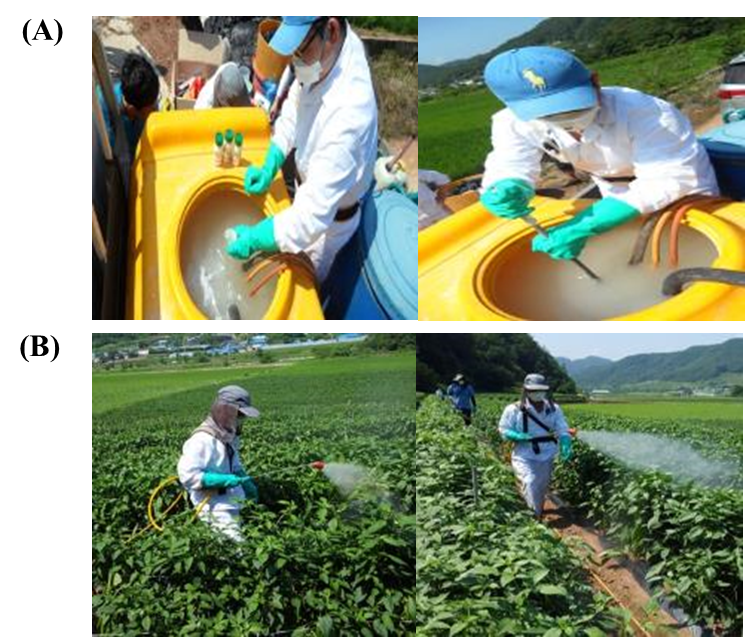
**

**Fig. S2.** Exposure scenarios of mix/load (A) and application (B).
